# Ventricular Tachycardia in the Absence of Structural Heart Disease

**Published:** 2005-04-01

**Authors:** Komandoor Srivathsan, Steven J Lester, Christopher P Appleton, Luis RP Scott, Thomas M Munger

**Affiliations:** 1 Division of Cardiovascular Diseases, Mayo Clinic, Rochester, Minnesota.; 2 Division of Cardiovascular Diseases, Mayo Clinic, Scottsdale, Arizona

**Keywords:** Ventricular tachycardia, structurally normal heart

## Abstract

In up to 10% of patients who present with ventricular tachycardia (VT), obvious structural heart disease is not identified. In such patients, causes of ventricular arrhythmia include right ventricular outflow tract (RVOT) VT, extrasystoles, idiopathic left ventricular tachycardia (ILVT), idiopathic propranolol-sensitive VT (IPVT), catecholaminergic polymorphic VT (CPVT), Brugada syndrome, and long QT syndrome (LQTS). RVOT VT, ILVT, and IPVT are referred to as idiopathic VT and generally do not have a familial basis. RVOT VT and ILVT are monomorphic, whereas IPVT may be monomorphic or polymorphic. The idiopathic VTs are classified by the ventricle of origin, the response to pharmacologic agents, catecholamine dependence, and the specific morphologic features of the arrhythmia. CPVT, Brugada syndrome, and LQTS are inherited ion channelopathies. CPVT may present as bidirectional VT, polymorphic VT, or catecholaminergic ventricular fibrillation. Syncope and sudden death in Brugada syndrome are usually due to polymorphic VT. The characteristic arrhythmia of LQTS is torsades de pointes. Overall, patients with idiopathic VT have a better prognosis than do patients with ventricular arrhythmias and structural heart disease. Initial treatment approach is pharmacologic and radiofrequency ablation is curative in most patients. However, radiofrequency ablation is not useful in the management of inherited ion channelopathies. Prognosis for patients with VT secondary to ion channelopathies is variable. High-risk patients (recurrent syncope and sudden cardiac death survivors) with inherited ion channelopathies benefit from implantable cardioverter-defibrillator placement. This paper reviews the mechanism, clinical presentation, and management of VT in the absence of structural heart disease.

## Introduction

It is estimated that 10% of patients who present with ventricular tachycardia (VT) have no obvious structural heart disease [1]. An absence of structural heart disease is usually suggested if an electrocardiogram (ECG) (except in Brugada syndrome and long QT syndrome [LQTS]), echocardiogram, and coronary arteriogram collectively are normal [2]. However, structural abnormalities may be identified by magnetic resonance imaging (MRI) even if all other test results are normal [3]. In addition, focal dysautonomia in the form of localized sympathetic denervation has been reported in patients with VT and no other obvious structural heart disease [4].

Types of VT that occur in the absence of structural heart disease include right ventricular (RV) monomorphic extrasystoles, RV outflow tract (RVOT) VT, left ventricular (LV) outflow tract (LVOT) VT, idiopathic LV tachycardia (ILVT), idiopathic propranolol-sensitive (automatic) VT (IPVT), catecholaminergic polymorphic VT (CPVT), Brugada syndrome, and LQTS. RV monomorphic extrasystoles, RVOT VT, LVOT VT, ILVT, and IPVT are referred to as idiopathic VT. Idiopathic VT from the RVOT and LV are monomorphic and generally not familial. Idiopathic VTs are classified with respect to the ventricle of origin, the response to pharmacologic agents, evidence of catecholamine dependence, and the specific morphologic features (QRS morphology, axis, pattern, and whether tachycardia is repetitive, nonsustained, or sustained) (Table 1). CPVT, Brugada syndrome, and LQTS are inherited ion channelopathies.

In this review of VT in the absence of structural heart disease, we discuss the clinical recognition and management of idiopathic VT and inherited ion channelopathies. The articles were selected for review from a search of PubMed using search terms “idiopathic VT,” “LQTS,” “Brugada syndrome,” and “CPVT.” For each topic, articles focusing on diagnosis and management were preferentially selected.

## RV Monomorphic Extrasystoles and RVOT VT

RV monomorphic extrasystoles and RVOT VT appear to be on a continuum of the same process. RV monomorphic extrasystoles are characterized by ventricular ectopy with left bundle branch block (LBBB) morphology, and, on ECG, the QRS axis is directed inferiorly. Ventricular ectopy of this type was defined in 1969 and considered “typical for normal subjects” [5]. Resting ECG in these patients has no identifiable abnormalities, and the prognosis is generally benign. These extrasystoles occur more often during the day than at night and are transiently suppressed by sinus tachycardia [6]. The extrasystoles may diminish or disappear with exercise during stress testing. The site of origin is most often the RVOT and, to a lesser extent, the interventricular septum in the region of the RVOT [7]. An echocardiogram is normal in most of these patients [8], although anatomic changes, such as focal thinning and fatty replacement of the RVOT, have been demonstrated with MRI [3].

A potential relationship between these seemingly benign ventricular extrasystoles of RVOT origin and structural cardiac disease (arrhythmogenic RV dysplasia [ARVD]) has been investigated.9 Sixty-one patients with RVOT ventricular extrasystoles were contacted 15 years after their initial visit; no patient had died of sudden death nor developed ARVD in this study. Two-thirds of the patients were asymptomatic, and, in half, the ventricular ectopy had disappeared. Focal fatty replacement of the RV was present on MRI in most patients, in contrast to the diffuse pattern of fatty replacement observed in patients with ARVD.

In North America, 70% of cases of idiopathic VT arise from the RV, chiefly the RVOT just inferior to the pulmonic valve [2]. The characteristic morphology of RVOT VT is a wide QRS complex tachycardia with LBBB pattern and an inferior axis [10]. Among the outflow tract tachycardias, 90% originate from the RVOT and 10% from the LVOT. Either or both forms may be found in the same patient. RVOT VT is usually diagnosed in the third to fifth decade of life, although cases at the extremes of age have been reported. Most patients (80%) present with palpitations or presyncope (50%) but rarely present with frank syncope. Exercise or emotional stress usually precipitates the tachycardia. Sudden death is rare.

**Figure 1:**
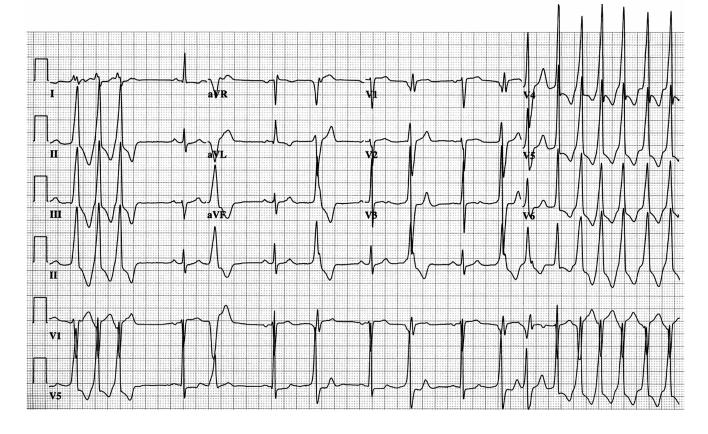
Electrocardiogram illustrating repetitive monomorphic right ventricular outflow tract ventricular tachycardia, left bundle branch block morphology, and an inferior axis.

**Figure 2:**
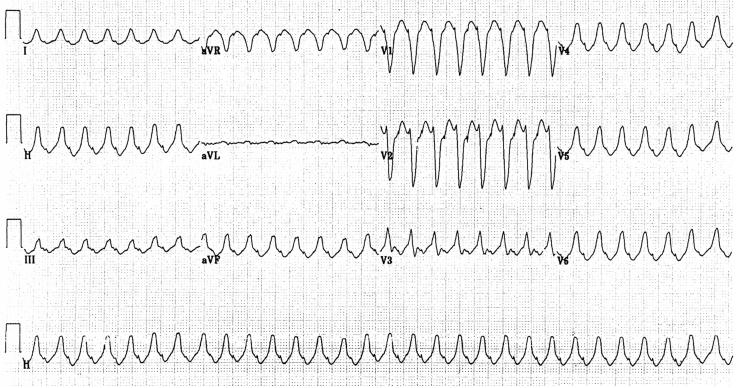
Electrocardiogram illustrating sustained right ventricular outflow tract ventricular tachycardia, left bundle branch block morphology, and an inferior axis.

**Figure 3:**
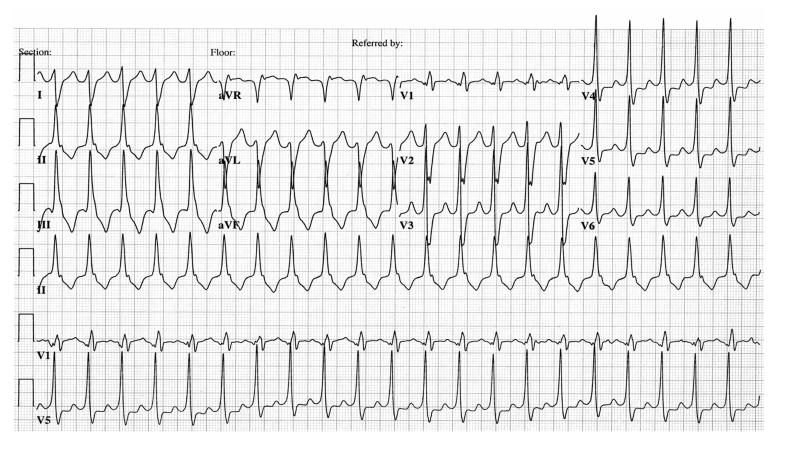
Electrocardiogram illustrating left ventricular outflow tract ventricular tachycardia (LVOT VT). The S wave in LI and R-wave transition in V1 suggest LVOT VT. In addition, an R:S amplitude ratio of 30% and an R:QRS duration ratio of 50% are seen. Presence of an S wave in leads V5 and V6 suggests an infravalvular origin of the tachycardia.

Two phenotypic forms of RVOT VT occur: nonsustained, repetitive, monomorphic VT (Figure 1) [11] and paroxysmal, exercise-induced, sustained VT (Figure 2). Both are terminated by the administration of adenosine. Specific ECG characteristics have been described to differentiate RVOT VT from LVOT VT [12]. LVOT VT may originate from either the supravalvular region of a coronary cusp or the infravalvular endocardial region of a coronary cusp of the aortic valve. The distinction between supravalvular and infravalvular location of the tachycardia has important therapeutic implications, particularly if radiofrequency (RF) ablation is performed. LVOT VT is suggested if the ECG during VT shows an S wave in lead I and an R-wave transition in lead V1 or V2. The absence of an S wave in V5 or V6 suggests a supravalvular location, whereas an S wave in leads V5 and V6 indicates an infravalvular location (Figure 3). In addition, in leads V1 and V2, an R:S amplitude ratio of 30% or more or an R:QRS duration ratio of 50% or more suggests an LV (aortic sinus cusp) origin of the tachycardia [13].

Exercise stress testing is used frequently to initiate and evaluate RVOT VT (unlike RV monomorphic extrasystoles, which are suppressed by sinus tachycardia) but is not clinically helpful in most cases. Initiation of the tachycardia depends on a critical heart rate that differs in each patient. The VT may be initiated during exercise or recovery [14]. ECG and echocardiogram in sinus rhythm are usually normal, as is coronary angiography. MRI may show abnormalities of the RV in up to 70% of patients, including focal thinning, diminished systolic wall thickening, and abnormal wall motion [15].

**Figure 4:**
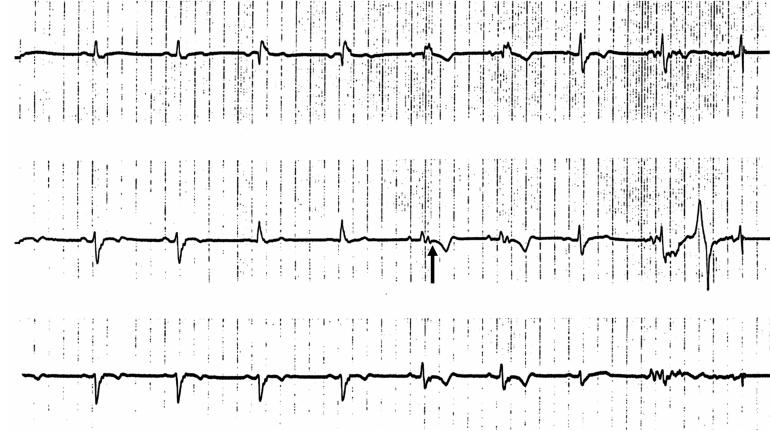
Electrocardiogram showing an epsilon wave (arrow) in a patient with arrhythmogenic right ventricular dysplasia.

RVOT VT should be distinguished from ARVD, a disorder with a more serious clinical outcome. The VT in ARVD may have morphologic features similar to RVOT VT (LBBB with inferior axis) but does not terminate with adenosine. In ARVD, the resting 12-lead ECG typically shows inverted T waves in right precordial leads. When present, RV conduction delay with an epsilon wave (Figure 4), best seen in leads V1-V2, is helpful in the diagnosis of ARVD. Measurement of serum brain natriuretic peptide may help distinguish ARVD from RVOT VT [16]. The level of brain natriuretic peptide is increased in ARVD, most likely due to increased expression by the surviving myocytes surrounded by atrophic tissue, which is indicative of the severity of RV dysfunction. Mechanisms of ARVD-related VT include both reentry facilitated by slow conduction through areas of fatty infiltration and increased automaticity [17]. In ARVD, the areas typically affected on echocardiography or MRI include the apex, interventricular septum below the tricuspid septal leaflet, and the RVOT. In some cases, fatty infiltration of the LV occurs. An RV biopsy and histopathologic characterization may help determine the correct diagnosis.

The differential diagnosis of RVOT VT also includes tachycardias associated with atriofascicular fibers (Mahaim fibers), atrioventricular reentrant tachycardia using a right-sided accessory pathway, and VT occurring in patients after repair of tetralogy of Fallot.

### Mechanism of RVOT VT

Intracellular calcium overload appears to be the principal underlying mechanism of RVOT VT. Cytosolic calcium overload enhances the function of the Na+/Ca2+ exchanger, which leads to increasing inward current and delayed afterdepolarization. When the inward current is of sufficient threshold, the delayed afterdepolarization may cause another action potential and initiate tachycardia. Cyclic adenosine monophosphate (cAMP) has a substantial role in regulating intracellular calcium. When the concentration of cAMP is increased, intracellular calcium levels are high. Adenosine is effective in terminating RVOT VT because of its ability to lower cAMP concentration [18]. Beta-blockers are often effective because of their inhibition of adenylate cyclase, which leads to a decrease of cAMP. Verapamil inhibits L-type calcium channels, which decreases the concentration of intracellular calcium and thereby has salutary effects.

Triggered activity, rather than reentry or enhanced automaticity, as the cause of RVOT VT is evidenced by termination with administration of adenosine and inability to entrain. The tachycardia may be inducible by programmed extrastimuli or by burst pacing the ventricle or atrium or by infusion of isoproterenol. Somatic mutation involving the G-protein signaling cascade could give rise to RVOT VT by disrupting adenosine signaling. Of interest, mutation of the G protein subunit Alphai2 has been identified on myocardial biopsy in only the RVOT (the site of origin) and not in myocardium remote from the site of VT [19].

### Treatment of RVOT VT

Acute termination of RVOT VT can be achieved by vagal maneuver or intravenous administration of adenosine, 6 mg, which can be titrated up to 24 mg as needed. Intravenous verapamil, 10 mg, given over 1 minute is an alternative, provided the patient has adequate blood pressure and has a previously established diagnosis of verapamil-sensitive VT. Lidocaine also may be effective in some cases. Hemodynamic instability warrants emergent cardioversion.

Long-term treatment options for RVOT VT include medical therapy or RF ablation. Medications, including beta-blockers or verapamil (diltiazem is equally effective), have a 25% to 50% rate of efficacy [20]. Alternative therapy includes class IA, class IC, and class III agents including amiodarone [20]. RF ablation now has cure rates of 90%, [10] which makes it a preferable option, given the young age of patients with RVOT VT. Ablation of sites at the aortic sinus cusp has been successful for treatment of LVOT VT [13], but serious complications may occur, including left main coronary artery occlusion. Coronary arteriography before and during ablation is recommended to keep the tip of the ablation catheter 1 cm away from the ostia of the coronary arteries. After ablation, arteriography should be repeated to assess the patency of coronary arteries. Epicardial foci of the LVOT remain a challenging ablation target.

## ILVT

Most VTs of LV origin are verapamil-sensitive intrafascicular tachycardias. Intrafascicular tachycardia has a right bundle branch block (RBBB) left-axis configuration in 90% to 95% of cases (exit site, left posterior fascicle) and the rest have RBBB with a right-axis pattern (exit site, left anterior fascicle). This form of VT is seen in the second to fourth decade of life and occurs more often in men (60%-80%) [21]. Symptoms during tachycardia include palpitations, dizziness, presyncope, and syncope. Sudden death is usually not seen, but one possible case has been reported [22].

A proposed diagnostic triad of ILVT includes: 1) induction with atrial pacing, 2) RBBB with left axis configuration, and 3) no evidence of structural heart disease [23]. A fourth feature, verapamil sensitivity, has since been described [24].

### Mechanism of ILVT

Focal reentry appears to be the principal mechanism of ILVT. The tachycardia cycle length can be increased with the administration of verapamil [25]. Some evidence implicates the Purkinje fibers of the fascicle as the area of slow conduction because of the presence of high-frequency potentials (Purkinje potentials) [26]. Others have found late diastolic potentials near the main trunk of the left bundle branch. Another hypothesis implicates a false tendon extending from the inferoposterior aspect of the LV to the basal septum as directly or indirectly having a role in causing this arrhythmia [27].

### Treatment of ILVT

In the acute setting, this tachycardia responds to intravenous verapamil. Termination with adenosine is rare, except for cases in which isoproterenol is used for induction of the tachycardia. Long-term therapy with verapamil is useful in mild cases and RF ablation is highly effective (85%-90%) in those with severe symptoms [21]. Identifying the focus of ablation may involve recognition of Purkinje potential, late diastolic potential, or earliest ventricular activation. Electroanatomic mapping may help localize the area of slow conduction [28]. In about 10% of cases of both ILVT and RVOT VT, a tachycardia with a different morphology may be inducible after successful ablation of clinical VT. This second tachycardia may be a cause for recurrence and should preferably be ablated during the initial attempt [29].

## IPVT

This form of idiopathic VT usually occurs by the fifth decade of life and can arise from the LV or RV [21]. The morphology of the tachycardia may be monomorphic or polymorphic. IPVT is not inducible with programmed stimulation. Isoproterenol infusion usually induces this VT. Beta-blockers are effective in terminating the tachycardia.

### Treatment of IPVT

Beta-blockers are used to treat this form of VT because they are effective in acute situations. There is insufficient information available regarding long-term management of IPVT. Survivors of sudden cardiac death may receive an implantable cardioverter-defibrillator (ICD).

## Inherited Channelopathies

### CPVT

CPVT is characterized by a uniform pattern of bidirectional polymorphic VT that can be easily and reproducibly induced during exercise or catecholamine infusion. A third of patients with CPVT have a family history of premature sudden death or stress-related syncope [30]. Exercise or acute emotion usually triggers syncope. Symptoms typically manifest in childhood; onset in adulthood has been reported but is uncommon.

The ryanodine receptor 2 (RyR2) is important for the regulation of intracellular calcium fluxes [31]. In patients with CPVT, the RyR2 gene is mutated, with autosomal dominant inheritance suggested [32]. One family with recessive CPVT has been reported, and the gene responsible produces the protein calsequestrin, which is functionally related to RyR2 [33].

CPVT and IPVT can be distinguished by means of family history, morphology (CPVT is usually bidirectional), age of onset (childhood vs the fifth decade), and in some cases genetic testing (genetic defect vs idiopathic).

#### Treatment

Beta-blockers are the preferred therapy for CPVT [30]. Beta-blockers may prevent syncope and sudden death because adrenergic activation is the main mechanism of delayed after depolarization-dependent triggered activity in these patients [34]. An ICD is required in 30% of patients because of symptomatic recurrence of life-threatening arrhythmia in spite of beta-blocker therapy [35].

### Brugada Syndrome

**Figure 5:**
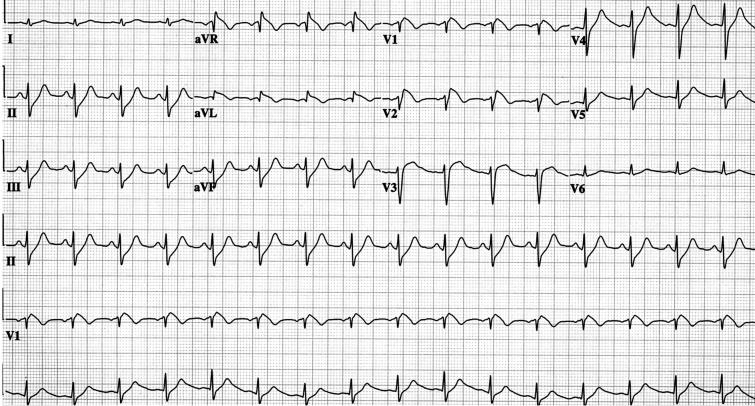
Electrocardiogram of a patient with Brugada syndrome. The right bundle branch block pattern with coved ST segment elevation (J-point elevation) is more than 2 mm, particularly in lead V2.

Brugada syndrome is characterized by apparent RBBB with ST elevation in V1 to V3 (V2 always present) (Figure 5), life-threatening cardiac arrhythmia (polymorphic VT) with no demonstrable structural cardiac disease, and familial occurrence [36,37]. The ECG changes may mimic acute myocardial infarction. The ECG findings may not be evident on resting 12-lead ECG but may be unmasked by flecainide or procainamide [38,39]. Two different types of ST elevation have been described: coved and saddleback. The coved type is more relevant to the syndrome than is the saddleback type [40]. Genetic analysis indicates that Brugada syndrome is due to mutation of the SCN5A protein [41]. The incidence of the disease is about 5 per 10,000 persons. The Brugada-type ECG (“Brugada sign”) may be much more common than is the clinical syndrome [42]. Sudden death is usually due to polymorphic VT or ventricular fibrillation. The disease predominantly affects young males.

The risk of sudden cardiac death with Brugada syndrome is substantial. In a study of 334 patients with typical Brugada-type ECG findings, which included symptomatic (cardiac arrest and syncope) and asymptomatic patients, the risk of recurrent events during 4 years of follow-up was 62% for those with cardiac arrest and 19% for those with syncope [37]. The asymptomatic group had an 8% event rate during 2 years of follow-up.

#### Management

ICD placement is the treatment of choice in symptomatic patients. Asymptomatic patients with Brugada-type ECG results should undergo electrophysiologic testing. If ventricular arrhythmia is inducible (two-thirds of patients are noninducible) the patient should receive an ICD. Asymptomatic patients with normal baseline ECG do not require further testing.

## LQTS

LQTS is an uncommon disorder in the general population. It is an inherited disorder, and mutations in 7 genes for LQTS have been identified to date (Table 2) [43]. This syndrome was initially identified in a family in which several children had syncope and sudden death. A recessive inheritance pattern was identified, and the syndrome was associated with deafness (Jervell and Lange-Nielsen syndrome). A similar and more common disorder without deafness, inherited in an autosomal dominant pattern, was subsequently identified (Romano-Ward syndrome).

### Clinical Diagnosis

Syncope, sudden cardiac death, or family screening of an affected individual is the reason that physicians evaluate patients for LQTS. Prolonged QT interval on ECG makes a diagnosis of LQTS likely. Medications that prolong QT interval must be carefully excluded from the patient’s medication list. Family history may be helpful for diagnosis of LQTS. An incidental finding of prolonged QTc (not due to medications) in an asymptomatic person is rare. Syncope and sudden death with LQTS occur with higher frequency during adolescence.

### Triggers of Clinical Events

In patients with LQT1 subtype, exercise seems to precipitate clinical events [44]. In those with LQT2, acute arousal, such as a sudden loud noise, tends to be a precipitating factor. In patients with LQT3, clinical events occur at rest or during sleep.

### Clinical Course

The risk of cardiac events is higher with certain genotypes; patients with LQT1 and LQT2 have higher risk of events than do those with LQT3 [45]. The risk of events also is higher during adulthood in females and during adolescence (before puberty) in males. The length of the QTc interval and the number of mutations also increase the risk. Once a clinical event occurs (syncope or survival after sudden cardiac death), recurrence is frequent.

### ECG Findings

Eighty percent of LQT1 and LQT2 carriers and 65% of LQT3 carriers have typical ECGs. In LQT1, the T wave is broad-based with an indistinct onset. In LQT2, bifid T waves may be seen in all 12 leads, and the ECG in LQT3 may have a long isoelectric ST segment [46].

### Treatment

Adrenergic modulation with beta-blockers is the most useful therapy in both symptomatic and asymptomatic patients, even though beta-blockers do not alter QTc interval [47]. However, the benefits of beta-blockers have not been proven in a randomized trial. Surgical sympathectomy is an adjuvant treatment and has been done rarely since the introduction of beta-blockers. Oral potassium may be useful in certain genotypes [48]. ICD placement, along with beta-blocker therapy, offers the best protection in high-risk patients (survivors of sudden death and those with recurrent syncope) [49].

## Acquired LQTS and Torsades de Pointes

Acquired prolongation of QT interval and pause-dependent, early afterdepolarization-mediated torsades de pointes most often is caused by medication and occasionally is caused by metabolic derangement (hypokalemia and hypomagnesemia) [50]. Correction of electrolyte abnormalities and discontinuation of precipitating drugs usually lead to amelioration of the arrhythmia. Intravenous magnesium, although it may not have an effect on QT interval, is highly effective at suppressing torsades de pointes. Occasionally, a temporary transvenous pacemaker or isoprenaline may be needed for effective management.

## Diagnostic Approach to Ventricular Arrhythmia in the Absence of Structural Heart Disease

**Figure 6:**
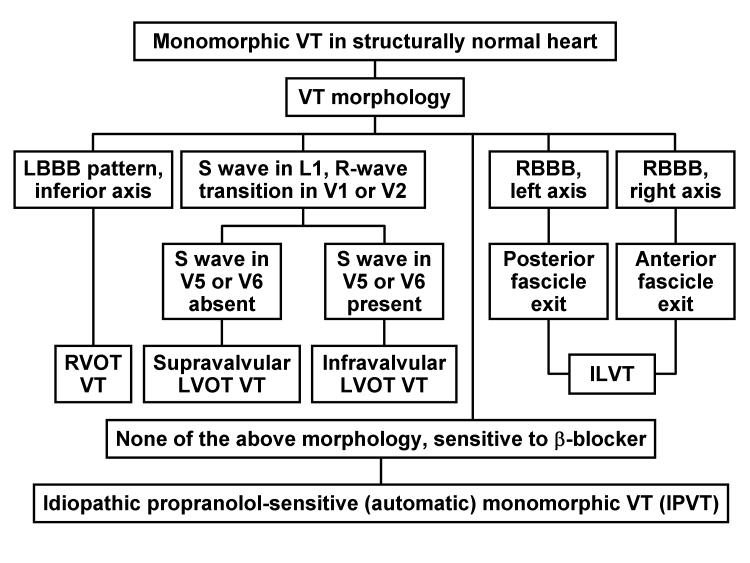
Diagnostic scheme for monomorphic ventricular tachycardia in structurally normal hearts (collectively normal electrocardiogram, echocardiogram, and coronary angiogram). ILVT, idiopathic left ventricular tachycardia; LBBB, left bundle branch block; LVOT, left ventricular outflow tract; RBBB, right bundle branch block; RVOT, right ventricular outflow tract; VT, ventricular tachycardia.

**Figure 7:**
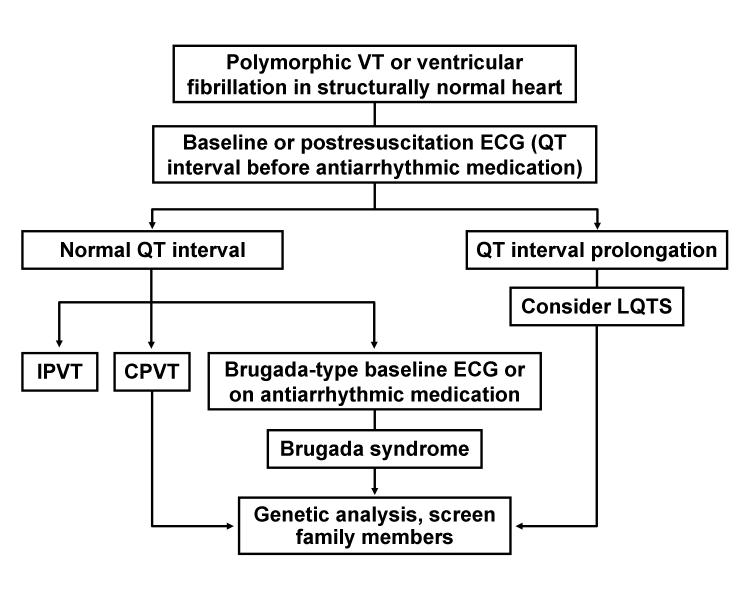
Proposed diagnostic scheme for polymorphic VT or ventricular fibrillation in structurally normal hearts (collectively normal electrocardiogram [except in Brugada syndrome and LQTS], echocardiogram, and coronary angiogram). CPVT, catecholaminergic polymorphic ventricular tachycardia; ECG, electrocardiogram; IPVT, idiopathic propranolol-sensitive (automatic) ventricular tachycardia; LQTS, long QT syndrome; VT, ventricular tachycardia.

The approach to diagnosis of the subtypes of VT in the absence of heart disease depends on the morphology of the tachycardia precipitating the clinical event. If the presentation is monomorphic VT, RVOT VT, LVOT VT, ILVT, and, rarely, IPVT are in the differential diagnosis. Specific ECG criteria should help clarify the diagnosis (Figure 6). If the inciting clinical event is precipitated by polymorphic VT, torsades de pointes, or ventricular fibrillation, the diagnosis may be approached by evaluating the baseline or postresuscitation QTc interval (without antiarrhythmic medications) (Figure 7). If the QTc is prolonged, LQTS and its subtypes are the predominant diagnoses, provided drug-induced QTc prolongation can be reasonably excluded. If the QTc is normal, Brugada syndrome, CPVT, and IPVT are the conditions in the differential diagnosis. The Brugada ECG, either at baseline or on induction with antiarrhythmic medication, may help identify Brugada syndrome. Bidirectional VT and presentation during childhood identify most patients with CPVT. IPVT remains a diagnosis when all other causes are unlikely and the arrhythmia is propranolol sensitive. Genetic testing may be helpful in the long-term evaluation of polymorphic VT but acutely is not helpful.

## Conclusion

Ventricular arrhythmia in the absence of structural heart disease is a small subset in the clinical spectrum of patients with VT. Overall, the prognosis is better in patients with idiopathic ventricular arrhythmia than in patients with structural heart disease and VT. Prognosis in hereditary channelopathies is variable; CPVT, in particular, has a malignant course when untreated. Understanding the different characteristics of these tachycardias, their diagnostic features, and physiologic substrates is essential for successful therapy and management. Stress testing and response to antiarrhythmics each have an important role in identifying the specific arrhythmia. RF ablation and placement of an ICD are important in the overall management of specific arrhythmia.

## Figures and Tables

**Table 1:**
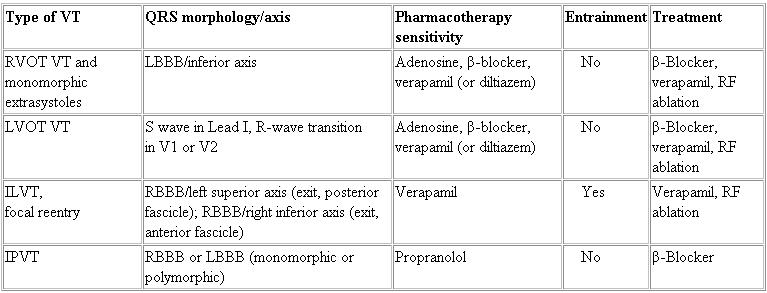
Types of Idiopathic Ventricular Tachycardia

ILVT, idiopathic left ventricular tachycardia; IPVT, idiopathic propanolol-sensitive ventricular tachycardia; LBBB, left bundle branch block; LVOT, left ventricular outflow tract; RBBB, right bundle branch block; RF, radiofrequency; RVOT, right ventricular outflow tract; VT, ventricular tachycardia.

**Table 2:**
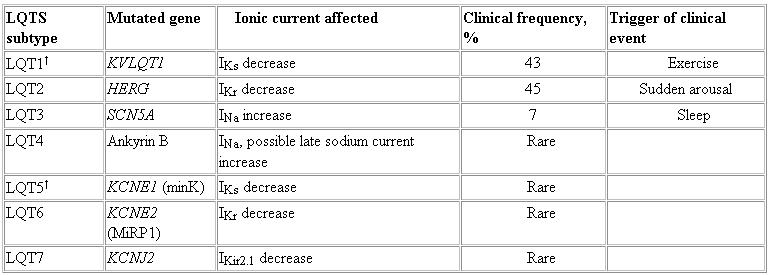
Genetic Subtypes of Long QT Syndrome*

I_Kir2.1_, inward rectifier K^+^ current; I_Kr_, rapid delayed rectifier K^+^ current; I_Ks_, slow delayed rectifier K^+^ current; LQTS, long QT syndrome. *Includes LQT1-LQT7 of autosomal dominant inheritance and Romano-Ward syndrome. †Homozygous mutations result in Jervell and Lange-Nielsen syndrome.

## Abbreviations

ARVD: arrhythmogenic right ventricular dysplasia
cAMP: cyclic adenosine monophosphate
CPVT: catecholaminergic polymorphic ventricular tachycardia
ECG: electrocardiogram
ICD: implantable cardioverter-defibrillator
ILVT: idiopathic left ventricular tachycardia
IPVT: idiopathic propranolol-sensitive (automatic) ventricular tachycardia
LBBB: left bundle branch block
LQTS: long QT syndrome
LV: left ventricle
LVOT: left ventricular outflow tract
MRI: magnetic resonance imaging
RBBB: right bundle branch blockk
RF: radiofrequency
RV: right ventricle
RVOT: right ventricular outflow tract
RyR2: ryanodine receptor
VT: ventricular tachycardia

## References

[R1] Klein LS, Shih HT, Hackett FK (1992). Radiofrequency catheter ablation of ventricular tachycardia in patients without structural heart disease. Circulation.

[R2] Miles WM (2001). Idiopathic ventricular outflow tract tachycardia: where does it originate?. J Cardiovasc Electrophysiol.

[R3] Carlson MD, White RD, Trohman RG (1994). Right ventricular outflow tract ventricular tachycardia: detection of previously unrecognized anatomic abnormalities using cine magnetic resonance imaging. J Am Coll Cardiol.

[R4] Mitrani RD, Klein LS, Miles WM (1993). Regional cardiac sympathetic denervation in patients with ventricular tachycardia in the absence of coronary artery disease. J Am Coll Cardiol.

[R5] Rosenbaum MB (1969). Classification of ventricular extrasystoles according to form. J Electrocardiol.

[R6] Leclercq JF, Rosengarten MD, Attuel P (1981). Idiopathic ventricular extrasystole: right ventricular parasystole not protected from the sinus rhythm?. Arch Mal Coeur Vaiss.

[R7] Coumel P, Leclercq JF, Slama R, Zipes DP, Jalife J (1985). Repetitive monomorphic idiopathic ventricular tachycardia. Cardiac electrophysiology and arrhythmias.

[R8] Proclemer A, Basadonna PT, Slavich GA (1997). Cardiac magnetic resonance imaging findings in patients with right ventricular outflow tract premature contractions. Eur Heart J.

[R9] Gaita F, Giustetto C, Di Donna P (2001). Long-term follow-up of right ventricular monomorphic extrasystoles. J Am Coll Cardiol.

[R10] Lerman BB, Stein KM, Markowitz SM (2000). Ventricular arrhythmias in normal hearts. Cardiol Clin.

[R11] Buxton AE, Marchlinski FE, Doherty JU (1984). Repetitive, monomorphic ventricular tachycardia: clinical and electrophysiologic characteristics in patients with and patients without organic heart disease. Am J Cardiol.

[R12] Hachiya H, Aonuma K, Yamauchi Y (2000). Electrocardiographic characteristics of left ventricular outflow tract tachycardia. Pacing Clin Electrophysiol.

[R13] Ouyang F, Fotuhi P, Ho SY (2002). Repetitive monomorphic ventricular tachycardia originating from the aortic sinus cusp: electrocardiographic characterization for guiding catheter ablation. J Am Coll Cardiol.

[R14] Gill JS, Prasad K, Blaszyk K (1998). Initiating sequences in exercise induced idiopathic ventricular tachycardia of left bundle branch-like morphology. Pacing Clin Electrophysiol.

[R15] Globits S, Kreiner G, Frank H (1997). Significance of morphological abnormalities detected by MRI in patients undergoing successful ablation of right ventricular outflow tract tachycardia. Circulation.

[R16] Matsuo K, Nishikimi T, Yutani C (1998). Diagnostic value of plasma levels of brain natriuretic peptide in arrhythmogenic right ventricular dysplasia. Circulation.

[R17] Pertsov AM, Davidenko JM, Salomonsz R (1993). Spiral waves of excitation underlie reentrant activity in isolated cardiac muscle. Circ Res.

[R18] Lerman BB, Belardinelli L, West GA (1986). Adenosine-sensitive ventricular tachycardia: evidence suggesting cyclic AMP-mediated triggered activity. Circulation.

[R19] Lerman BB, Dong B, Stein KM (1998). Right ventricular outflow tract tachycardia due to a somatic cell mutation in G protein subunitai2. J Clin Invest.

[R20] Buxton AE, Waxman HL, Marchlinski FE (1983). Right ventricular tachycardia: clinical and electrophysiologic characteristics. Circulation.

[R21] Iwai S, Lerman BB (2000). Management of ventricular tachycardia in patients with clinically normal hearts. Curr Cardiol Rep.

[R22] German LD, Packer DL, Bardy GH (1983). Ventricular tachycardia induced by atrial stimulation in patients without symptomatic cardiac disease. Am J Cardiol.

[R23] Zipes DP, Foster PR, Troup PJ (1979). Atrial induction of ventricular tachycardia: reentry versus triggered automaticity. Am J Cardiol.

[R24] Belhassen B, Rotmensch HH, Laniado S (1981). Response of recurrent sustained ventricular tachycardia to verapamil. Br Heart J.

[R25] Tsuchiya T, Okumura K, Honda T (2001). Effects of verapamil and lidocaine on two components of the re-entry circuit of verapamil-sensitive idiopathic left ventricular tachycardia. J Am Coll Cardiol.

[R26] Nakagawa H, Beckman KJ, McClelland JH (1993). Arrhythmias/Electrophysiology/Pacing/ECGS: radiofrequency catheter ablation of idiopathic left ventricular tachycardia guided by a Purkinje potential.. Circulation.

[R27] Thakur RK, Klein GJ, Civaram CA (1996). Anatomic substrate for idiopathic left ventricular tachycardia. Circulation.

[R28] Betts TR, Roberts PR, Allen SA (2000). Radiofrequency ablation of idiopathic left ventricular tachycardia at the site of earliest activation as determined by noncontact mapping. J Cardiovasc Electrophysiol.

[R29] Lokhandwala YY, Vora AM, Naik AM (1999). Dual morphology of idiopathic ventricular tachycardia. J Cardiovasc Electrophysiol.

[R30] Leenhardt A, Lucet V, Denjoy I (1995). Catecholaminergic polymorphic ventricular tachycardia in children: a 7-year follow-up of 21 patients. Circulation.

[R31] Marks AR, Priori S, Memmi M (2002). Involvement of the cardiac ryanodine receptor/calcium release channel in catecholaminergic polymorphic ventricular tachycardia. J Cell Physiol.

[R32] Priori SG, Napolitano C, Tiso N (2001). Mutations in the cardiac ryanodine receptor gene (hRyR2) underlie catecholaminergic polymorphic ventricular tachycardia.. Circulation.

[R33] Lahat H, Eldar M, Levy-Nissenbau E (2001). Autosomal recessive catecholamine- or exercise-induced polymorphic ventricular tachycardia: clinical features and assignment of the disease gene to chromosome 1p13-21. Circulation.

[R34] Nakajima T, Kaneko Y, Taniguchi Y (1997). The mechanism of catecholaminergic polymorphic ventricular tachycardia may be triggered activity due to delayed afterdepolarization. Eur Heart J.

[R35] Priori SG, Napolitano C, Memmi M (2002). Clinical and molecular characterization of patients with catecholaminergic polymorphic ventricular tachycardia. Circulation.

[R36] Brugada P, Brugada J (1992). Right bundle branch block, persistent ST segment elevation and sudden cardiac death: a distinct clinical and electrocardiographic syndrome: a multicenter report. J Am Coll Cardiol.

[R37] Brugada P, Brugada R, Brugada J (2000). Sudden death in patients and relatives with the syndrome of right bundle branch block, ST segment elevation in the precordial leads V(1) to V(3) and sudden death. Eur Heart J.

[R38] Miyazaki T, Mitamura H, Miyoshi S (1996). Autonomic and antiarrhythmic drug modulation of ST segment elevation in patients with Brugada syndrome. J Am Coll Cardiol.

[R39] Krishnan SC, Josephson ME (1998). ST segment elevation induced by class IC antiarrhythmic agents: underlying electrophysiologic mechanisms and insights into drug-induced proarrhythmia. J Cardiovasc Electrophysiol.

[R40] Ikeda T (2002). Brugada syndrome: current clinical aspects and risk stratificaiton. Ann Noninvasive Electrocardiol.

[R41] Antzelevitch C, Brugada P, Brugada J (2002). Brugada syndrome: a decade of progress. Circ Res.

[R42] Littmann L, Monroe MH, Kerns WP (2003). Brugada syndrome and “Brugada sign”: clincial spectrum with a guide for the clinician. Am Heart J.

[R43] Moss AJ (2003). Long QT syndrome. JAMA.

[R44] Schwartz PJ, Priori SG, Spazzolini C (2001). Genotype-phenotype correlation in the long-QT syndrome: gene-specific trigger for life-threatening arrhythmias. Circulation.

[R45] Zareba W, Moss AJ, Schwartz PJ (1998). Influence of genotype on the clinical course of the long-QT syndrome. N Engl J Med.

[R46] Roden DM, Anderson ME (2000). The pause that refreshes, or does it? Mechanisms in torsades de pointes. Heart.

[R47] Moss AJ, Zareba W, Hall WJ (2000). Effectiveness and limitations of beta-blocker therapy in congenital long-QT syndrome. Circulation.

[R48] Compton SJ, Lux RL, Ramsey MR (1996). Genetically defined therapy of inherited long-QT syndrome: correction of abnormal repolarization by potassium. Circulation.

[R49] Zareba W, Moss AJ, Daubert JP (2003). Implantable cardioverter defibrillator in high-risk long QT syndrome patients. J Cardiovasc Electrophysiol.

[R50] Mansfield RJ, Thomas RD (2001). Recurrent syncope: drug induced long QT syndrome. Postgrad Med J.

